# Breastfeeding has long-term impacts on the brain and body, but where do we go from here? (Commentary on ‘Breastfeeding duration and brain-body development in 9–10-year-olds: modulating effect of socioeconomic levels’)

**DOI:** 10.1038/s41390-024-03533-5

**Published:** 2024-09-02

**Authors:** Brittany R. Howell

**Affiliations:** 1 Fralin Biomedical Research Institute at Virginia Tech Carilion, Virginia Tech, Roanoke, VA USA; 2 Department of Human Development and Family Science, Virginia Tech, Blacksburg, VA USA

Many investigators have sought to better understand the relationships between early infant feeding, specifically provision of human milk, on the brain and body, with particular focus on long-term outcomes. In a manuscript published in June of 2024, Rajagopalan and colleagues^1^ did just that, using data from more than 7500 participants from the Adolescent Brain and Cognitive Development (ABCD) cohort, specifically examining brain structure and body adiposity contextualized by socioeconomic environment. The ABCD dataset represents one of the richest cohort studies of child development ever collected, comprised of longitudinal brain imaging, physiological, cognitive, environmental, and sociodemographic measures collected from 9 to 10 years of age (baseline) and continuing across adolescence (through approximately age 18) in a diverse sample of nearly 12,000 individuals across the United States.^2^ As of the writing of this commentary the ABCD team has released 5 waves of data, which have yielded more than 1000 publications.^3^ While leveraging this unique resource to answer questions regarding long-term impacts of milk feeding on child development seems like an obviously worthy endeavor, it is not without caveats, requiring careful consideration of the findings presented.

Infant feeding is complex, with practices impacted by maternal health, local policy, sociocultural traditions, and family situations. In the United States this often leads to differences in feeding practice along socioeconomic and racial lines, with lower resourced and marginalized mothers on average providing mother’s milk to their infants for shorter periods of time.^4^ This was true for the sample used in the current study, with those providing human milk to their infants for longer durations having both higher family income and parental education. No analyses of potential main effects of the primary measure of socioeconomic environment (SEE) collected in ABCD, Area Deprivation Index, ADI, were presented, so it is unclear whether those in lower ADI (more resourced) environments had longer durations of breastfeeding. Also, of particular relevance for the adiposity findings presented by Rajagopalan and colleagues, breastfeeding outcomes are poorer in women with higher pre-pregnancy BMI.^5^ Unfortunately, maternal pre-pregnancy BMI was not collected as part of the ABCD dataset, so was not included in the analyses. These complexities make it very difficult to disentangle the direct impacts of milk feeding on the brain and body from the direct impacts of these other aspects of the infant’s environment, even in studies explicitly designed to address this major gap in knowledge. The approach taken by Rajagopalan et al further muddies the waters, as they group breastfeeding duration into 6-month bins, aren’t able to account for proportions of formula or human milk in mixed fed babies, or current dietary patterns, making it difficult to identify any specific potential sensitive periods or drivers of these effects. It is important to note that these bins are consistent with the current breastfeeding recommendations of both the World Health Organization (WHO) and the American Academy of Pediatrics (AAP),^6^ which does help to contextualize their recommendations. Given that ABCD was not intended to address these questions, and the other limitations highlighted above, it is very difficult to propose specific interventions based on these findings to improve the health of infants.

Many studies have connected histories of milk feeding with better long-term BMI outcomes across the lifespan;^7^ however, the evidence for positive impacts on brain development is much less consistent and compelling. This is in part due to a lack of understanding of what “good” or “optimal” brain development looks like, and flagrant conflation of cognitive measures and brain development (not to say that they are completely independent processes^8^) throughout the literature. Rajagopalan and co-authors report significant correlations between duration of breastfeeding and global measures of brain structure, including positive associations between cortical surface area, both cortical and subcortical gray matter volumes, and a negative association with cortical white matter volume. While these findings highlight the potential long-term impacts of human milk feeding, given the extensive literature describing the importance of many aspects of the environment in shaping the brain during infancy, childhood, and adolescence,^9^ these findings do not robustly support a specific role for breastfeeding in supporting long-term structural brain development. It is interesting to note the lack of a significant interaction between breastfeeding duration and SEE on the global brain measures assessed and adiposity. These results suggest that the environment, and the aspects of the environment captured by the ADI in particular, are not as salient to the developing brain and body as early exposure to human milk. The authors do present analyses stratified by ADI to explore differential impacts based on SEE; however, these analyses should be interpreted with caution given the lack of significant interactions reported and the demographic differences reported for participants who breastfed for longer durations. The authors attempt to address these differences in baseline demographics by incorporating many known confounders into their statistical models, including indicators of infant and maternal health, but unfortunately statistics do not eliminate sample bias.

Despite the limitations of the study presented, it is remarkable that there is still long-term impact, albeit a relatively small one, of breastfeeding duration on the brain and body. These findings combined with the limitations highlight the deep gaps in understanding of early feeding and subsequent health in adolescence, and suggest a clear path to the knowledge needed to design interventions and policies to support long-term health. This path includes focusing on engagement with more diverse communities where breastfeeding and environmental influencers (including SEE) may be better disentangled, designing research studies that incorporate measures of the early environment that are more impactful for infant brain development and adiposity than those included in the ADI, and working to define the underlying mechanisms of these effects, including better understanding of milk as a biological system and relevant cellular and molecular mechanisms.^10^ Prospective, longitudinal studies explicitly and intentionally designed by teams of scientists and clinicians are required to answer these important questions robustly. Until then we will be limited to appreciating that milk is a key driver of lifelong health, but we will be unable to effectively extend the application of those benefits to all infants.

This work also clearly supports the need for increased efforts to make it possible for all families to provide human milk to their infants. Efforts like the Momnibus Act^11^ are being pioneered by US Congressional Representatives and maternal and infant health advocates to build the infrastructure needed to ensure equitable and easy access to programs that would improve the breastfeeding environment, especially for working mothers. Infant health is a worthy investment, and work such as that presented by Rajagopalan and colleagues provides a great example of the importance of breastfeeding, particularly longer durations of breastfeeding, and the ability of early diet to program systems in the body that impact long-term health. Understanding the science of milk and brain and body development will help us to more effectively and specifically target optimal neurodevelopmental and metabolic outcomes, while providing further evidence of the importance of this ubiquitous human experience in lifespan health, ultimately providing the best start for all people.
